# Compositional and Functional Characteristics of Feta-Type Cheese Made from Micellar Casein Concentrate

**DOI:** 10.3390/foods11010024

**Published:** 2021-12-23

**Authors:** Ahmed R. A. Hammam, Rohit Kapoor, Prafulla Salunke, Lloyd E. Metzger

**Affiliations:** 1 Dairy and Food Science Department, South Dakota State University, Brookings, SD 57007, USA; prafulla.salunke@sdstate.edu (P.S.); Lloyd.Metzger@sdstate.edu (L.E.M.); 2 National Dairy Council, Rosemont, IL 60018, USA; rohit.kapoor@dairy.org

**Keywords:** micellar casein concentrate, Feta-type cheese, adjusted yield, hardness, rheological characteristics, cheese color

## Abstract

Micellar casein concentrate (MCC) is a high protein ingredient (obtained by microfiltration of skim milk) with an elevated level of casein as a percentage of total protein (TP) compared to skim milk. It can be used as an ingredient in cheese making. Feta-type cheese is a brined soft cheese with a salty taste and acid flavor. We theorize that Feta-type cheese can be produced from MCC instead of milk, which can improve the efficiency of manufacture and allow for the removal of whey proteins before manufacturing Feta-type cheese. The objectives of this study were to develop a process of producing Feta-type cheese from MCC and to determine the optimum protein content in MCC to make Feta-type cheese. MCC solutions with 3% (MCC-3), 6% (MCC-6), and 9% (MCC-9) protein were prepared and standardized by mixing water, MCC powder, milk permeate, and cream to produce a solution with 14.7% total solids (TS) and 3.3% fat. Thermophilic cultures were added at a rate of 0.4% to MCC solutions and incubated at 35 °C for 3 h to get a pH of 6.1. Subsequently, calcium chloride and rennet were added to set the curd in 20 min at 35 °C. The curd was then cut into cubes, drained for 20 h followed by brining in 23% sodium chloride solutions for 24 h. Compositional analysis of MCC solutions and cheese was carried out. The yield, color, textural, and rheological measurements of Feta-type cheese were evaluated. Feta-type cheese was also made from whole milk as a control. This experiment was repeated three times. The yield and adjusted yield of Feta-type cheese increased from 19.0 to 54.8 and 21.4 to 56.5, respectively, with increasing the protein content in MCC from 3% to 9%. However, increasing the protein content in MCC did not show significant differences in the hardness (9.2–9.7 kg) of Feta-type cheese. The color of Feta-type cheese was less white with increasing the protein content in MCC. While the yellowish and greenish colors were high in Feta-type cheese made from MCC with 3% and 6% protein, no visible differences were found in the overall cheese color. The rheological characteristics were improved in Feta-type cheese made from MCC with 6% protein. We conclude that MCC with different levels of protein can be utilized in the manufacture of Feta-type cheese.

## 1. Introduction

Feta cheese and Feta-type cheese are brined white soft cheeses made from goat and sheep’s milk, but these days, different types of milk, such as cow and buffalo milk, are also utilized [1,2]. Feta cheese and Feta-type cheese have a salty taste with an acid flavor. Although it was manufactured from raw milk to have the unique flavors obtained by natural microflora, it is currently produced from pasteurized milk to ensure food safety and to achieve a more standardized product in terms of its composition, functionality, and organoleptic properties. Feta cheese and Feta-type cheese can be produced from whole milk, partially skimmed, or skim milk [1,3,4]. The composition of Feta cheese and Feta-type cheese made from whole milk ranges from 45–60% moisture, 10–20% fat, 15–20% protein, 4.6–5.3 pH, and 5–10% salt [4,5,6].

There is an opportunity to use Micellar casein concentrate (MCC) instead of milk to produce Feta-type cheese. MCC is a high protein ingredient produced by microfiltration (MF) of skim milk using semi-permeable membranes with a 0.1 µm pore size. MCC can be produced in liquid or dried forms. The typical composition of liquid MCC using 3-stages MF with 3× concentration factor (CF) and diafiltration (DF) is >9% total protein (TP) and >13% total solids (TS) [7]. MCC has promising applications in several dairy and other food products due to its unique physicochemical and functional characteristics (e.g., foaming, emulsifying, wetting, dispersibility, heat stability, bland flavor, and water-binding ability) [8,9]. The high casein content in MCC makes it heat stable, and thereby. it can be used in beverages that require sterilization [10,11]. Non-dairy applications of MCC are pasta, confectionery, meat products, special dietary preparations, textured products, convenience foods, toothpaste, cosmetics, and wound treating preparations [8,12]. Dairy applications for MCC include Cheddar cheese [13,14,15], Greek-style yogurt [16], imitation Mozzarella cheese [12,17], recombined MCC [18,19], process cheese and process cheese products [7,20,21], and acid curd [7,21,22].

The liquid MCC can be used immediately in making Feta-type cheese or diluted to lower protein levels before making the cheese. Making Feta-type cheese from MCC has advantages compared to milk. A traditional Feta cheese produced from milk results in whey as a byproduct, which is more challenging to utilize. Starting with MCC may potentially allow for increased cheese-making efficiency due to a reduction in the whey stream [8,14]. The manufacturing of MCC using MF results in milk-derived whey protein and whey permeate (lactose-rich stream) as side streams, which can be further separated and used to make value-added products, such as native whey protein isolate, whey permeate, and lactose, respectively. These ingredients have recently been of great value to the dairy and food industry [8,23]. Therefore, if MCC is the starting material for making Feta-type cheese instead of milk, there is a potential for manufacturers to have added benefits as various values high-value side streams of MCC can be collected even before starting cheese making.

MCC is produced from skim milk, which will result in low-fat Feta-type cheese. As a result, the addition of fat to MCC before making Feta-type cheese is required to match the compositional and functional characteristics of Feta-type cheese made from whole milk. MCC can be fortified with different sources of fat, such as cream and vegetable oils [24,25]. A variety of Feta-type cheese from MCC can be obtained based on the source of fat. As a result, the manufacture of Feta-type cheese from MCC may attract a wide range of consumers. Thus far, no studies have presented data related to the application of MCC in the manufacture of Feta-type cheese. We hypothesize that MCC can be utilized in making high-quality Feta-type cheese with a higher yield compared to milk. The objectives of this study were to develop a process to produce Feta-type cheese using MCC and to evaluate the effect of protein content in the starting MCC solution on the functional properties and the yield of the final Feta-type cheese produced.

## 2. Materials and Methods

### 2.1. Preparation of MCC

In this study, MCC powder was reconstituted and standardized to have 3%, 6%, and 9% protein. Water, MCC powder 80% protein (Leprino Foods, Denver, CO 80211), pasteurized heavy cream from a local store (Land O’Lakes 40% fat, commercial market), and milk permeate powder (IdaPro milk permeate, Idaho milk products, Jerome, Idaho 83338) were utilized to prepare Feta-type cheese formulations (Table 1) using TechWizard software (Excel-based software Owl Software, Columbia, Missouri 65201) [26]. Milk permeate was used to standardize the solid’s content in the MCC formulations. All MCC formulations were standardized to have 3.3% fat and 14.7% TS. Since the typical MCC is manufactured from skim milk, the cream was added to the MCC formulations as a source of fat to match the fat content in whole bovine milk. All ingredients except cream were mixed thoroughly in 1 L beakers for approximately 2 h at room temperature using a magnetic stirrer (Fisher Scientific, CAT No 11-100-49SH). The solutions were then pasteurized at 65 °C for 30 min. The MCC solution was then transferred into 2 L sterilized plastic containers (Uline, Hudson, WI 54016). Subsequently, the MCC was cooled to 35 °C (inoculation temperature).

### 2.2. Manufacture of Feta-Type Cheese

Feta-type cheese was manufactured from 3%, 6%, and 9% protein MCC solutions. Concurrently, Feta-type cheese was also manufactured from whole bovine milk (Land O’Lakes) as a control. The cheese was made as described in previous studies [5,27,28] with some modifications. Thermophilic YoFlex cultures (*Lactobacillus delbrueckii* subsp. *bulgaricus* and *Streptococcus thermophilus*; DVS YF-L706, Batch No 3287760, Chr. Hansen, Hørsholm, Denmark) were added at a rate of 0.4% at 35 °C to get a pH of 6.1 (approximately 3 h). Subsequently, the cream was added and mixed thoroughly. A 10% calcium chloride solution (Food Grade, FBC Industries, Rochelle, IL 61068) was then added at a rate of 50 mL kg^−1^ milk. Then, rennet (CHY-MAX Extra, CHR Hansen, Batch No: 3429198, Material No: 73812) was added. The recommended dosage of rennet ranged from 30 to 60 IMCU L^−1^ milk so 45 IMCU L^−1^ milk was added. The rennet was diluted 10 times and added at a rate of 0.1% to coagulate the milk for 20 min at 35 °C. The pH of cheese curd was monitored every hour during cheese making using a Hannah pH meter (Hannah Edge Blu, Woonsocket, RI 02895). Subsequently, the cheese was cut into cubes, left for 20 min, and then filled into cheese molds equipped with cheesecloth to drain the whey. Weights of around 500 g were placed on the control and MCC-3 treatments, while 700 g were placed on MCC-6 and MCC-9 to drain the whey for 20 h. Subsequently, the cheese was further cut into blocks and brined in sterilized plastic containers with 23% of sodium chloride solution (*w*/*w*) and stored at 4 °C for 24 h. After that, cheese was removed from the salt solution for analysis. This study was repeated three times using three different batches of MCC.

### 2.3. Analyses

#### 2.3.1. Cheese Composition

Milk and MCC solutions used in making Feta-type cheese were analyzed for ash (AOAC, 2000; method 945.46; 33.2.10), TS (AOAC, 2000; method 990.20; 33.2.44) using an air draft oven, TP (AOAC, 2000; method 991.20; 33.2.11), and noncasein nitrogen (NCN) (AOAC, 2000; method 998.05; 33.2.64) using the Kjeldahl method [29]. The TP and NCN were calculated as nitrogen and multiplied by 6.38. The cheese was also analyzed for ash, TS, TP, fat, and salt. The fat of the final Feta-type cheese was measured using the Mojonnier method (AOAC, 2000; method 989.05; 33.2.26). The salt content was determined using a Chloride Analyzer 926 (Nelson-Jameson, Marshfield, WI 54449) with some modifications [30,31]. The final solution used to determine the salt content was diluted with distilled water in a ratio of 1:1 due to the high salt content. The yield and adjusted yield of Feta-type cheese were calculated using Equations (1) and (2), respectively [32,33]:

(1) $$\mathrm{Actual}\;\mathrm{yield}\;\left(\mathrm{AY}\right)=\left(\frac{\mathrm{Curd}\;\mathrm{weight}}{\mathrm{MCC}\;\mathrm{weight}}\right)\times 100$$

(2) $$\mathrm{Adjusted}\;\mathrm{yield}\;=\mathrm{AY}\times \left(\frac{100-\mathrm{actual}\%\mathrm{water}+\mathrm{actual}\%\mathrm{salt}}{100-\mathrm{desired}\%\mathrm{water}+\mathrm{desired}\%\mathrm{salt}}\right)$$

#### 2.3.2. Rheological Characteristics

The rheological characteristics of Feta-type cheese were measured using an MSR 92 rheometer (Anton Paar, Graz, Austria) as described in a previous study [34]. Strain sweep and frequency sweep tests were performed using 25 mm parallel plate geometry at 20 °C with a 1 mm gap size. The storage modulus (G’; measures elastic), loss modulus (G′′; measures viscous), and tan (δ) were monitored. The cheese samples were warmed at room temperature for 1 h before measuring the rheological characteristics. A small amount of cheese was placed on the lower plate, and then the upper plate was moved down until a 1 mm gap size was reached. The strain sweep test was performed to determine the linear viscoelastic region at a range of 0.01% to 2.0% strain and 0.1 Hz frequency. The frequency sweep test was measured after determining the linear viscoelastic region (0.02%) at a range of 0.1 to 100 Hz frequency. One hundred data points were collected in each test. The rheological properties were measured in duplicates.

#### 2.3.3. Hardness

The hardness of Feta-type cheese samples was evaluated using a TA.XT-Plus Texture Analyzer (TA.XT-Plus, 6 Patton Drive, South Hamilton, MA, USA) as described by Prasad and Alvarez [35]. The cheese samples were cut into cubes with dimensions 3 × 3 × 2 cm (L × W × H). The texture analyzer was equipped with a 38 mm diameter cylindrical flat probe (TA-4). The probe was calibrated at 20 mm height. The compression mode was set at 1 mm s^−1^ pre-test speed, 0.5 mm s^−1^ test speed, and 2 mm s^−1^ post-test speed with a distance of 10 mm and double bite compression. The maximum force of the first compression was referred to as the hardness of Feta-type cheese and reported in grams. Hardness analyses were performed in triplicate.

#### 2.3.4. Color Analysis

A Minolta Spectrophotometer (CM-508d, Minolta camera Co., Ltd., Osaka, Japan) was used to measure the color of the cheese [36,37]. The color test was performed in triplicate. A white standard board was used for calibration of the colorimeter before measuring the cheese color. The cheese color was represented in L*, a*, and b* values. The L* value has a range of 0 (black) to 100 (white). The a* value represents a range of −60 (green) to +60 (red), while the b* value has a range of −60 (blue) to +60 (yellow). The color difference (ΔΕ), which refers to the change in visual perception of colors, was also calculated as described in Power’s study using Equation (3) [36]:

(3) $$\Delta E\;=\sqrt{{\left(L_{0}-L\right)}^{2}+{\left(a_{0}-a\right)}^{2}+{\left(b_{0}-b\right)}^{2}}$$

### 2.4. Statistical Analysis

Statistical analysis was performed using R software (R × 64-3.3.3, R Foundation for Statistical Computing). All data were analyzed by ANOVA using GLM to analyze the differences among means (mean ± SD/SEM) for each variable. When significant differences were found at *p* < 0.05, mean separation was performed using the least significant difference test.

## 3. Results and Discussion

### 3.1. Composition

The composition of milk and MCC solutions (MCC-3, MCC-6, and MCC-9) utilized in making Feta-type cheese is presented in Table 2. The fat content in whole milk was approximately 3.3%, so the cream was added to have 3.3% fat content in all MCC solutions. Additionally, the protein was standardized to get 3%, 6%, and 9% TP. The TS of whole milk and MCC solutions is shown in Table 2. The TS in whole milk was 12.4%, while it was 14.7%, 14.6%, and 14.8% in MCC-3, MCC-6, and MCC-9, respectively. It was expected that the TS in MCC solutions would be high (*p* < 0.05) compared to the TS of milk. However, no significant difference (*p* > 0.05) was found in the TS within MCC solutions. The obtained TP in MCC solutions were close to the expected values and were significantly different (*p* < 0.05) within treatments, which were 3.3% in MCC-3, 6.4% in MCC-6, and 9.6% in MCC-9 compared to 3.0% in milk. Since we standardized only the solids, the TP:fat ratio was not similar (*p* < 0.05) in the MCC solutions used to make Feta-type cheese and this ratio increased with elevating the protein content in MCC solutions. However, the TP:fat ratio was not different (*p* < 0.05) in whole milk and MCC-3, which was around 1.0. This ratio increased to 1.9 and 2.9 MCC-6 and MCC-9 solutions, respectively. The ash content was approximately 0.8% in all MCC solutions compared to 0.7% in milk (*p* < 0.05). The NCN increased (*p* < 0.05) from approximately 0.6% to 0.9% with increasing TP content in MCC solutions from 3.0% to 9.0%, while the NCN in milk was 0.9%. It has been reported that the NCN increases as the protein content increases [38]. The composition of the 9% protein MCC solution was similar to the typical composition of liquid MCC reported in our previous studies [7,8], produced using three MF stages with 3× CF and DF.

**Figure 1:**
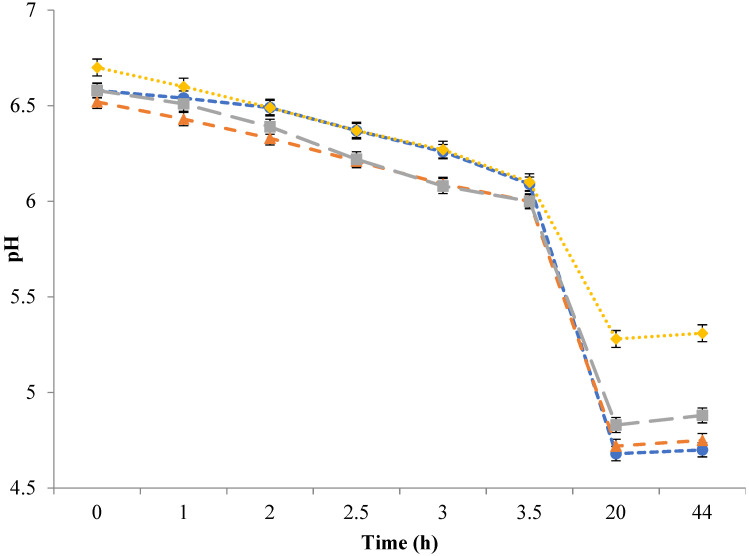
Mean (n = 3) pH values measured during the manufacture of Feta-type cheese from micellar casein concentrate (MCC). Treatment: Control (●) = Feta-type cheese made from whole milk; MCC-3 (▲) = Feta-type cheese made from MCC solution with 3% protein; MCC-6 (■) = Feta-type cheese made from MCC solution with 6% protein; MCC-9 (♦) = Feta-type cheese made from MCC solution with 9% protein.

The pHs of milk, MCC-3, MCC-6, and MCC-9 were monitored during the manufacture of Feta-type cheese (Figure 1). The pH of MCC solutions ranged from 6.5 to 6.7, while it was 6.6 for milk. The pH of MCC with 3% and 6% protein decreased to 6.1 after 3 h of inoculation with starter cultures, while the pH of milk and 9% protein MCC reached 6.1 after approximately 3.5 h; this could be due to the slight differences in the pH of initial material and lactose content.

The TS, TP, salt, ash, fat, and TP to fat ratios in Feta-type cheese made from milk and MCC with different protein contents are illustrated in Table 3. The TS of Feta-type cheese ranged from 44.7% to 50.5%. There was a slight difference in TS of final Feta-type cheese; however, no significant differences (*p* > 0.05) were detected. The TP of Feta-type cheese made from milk and MCC solutions were significantly different (*p* < 0.05). The Feta-type cheese made from 3% protein MCC had the lowest values of TP with 14.04%, while the highest TP content was found in Feta-type cheese made from MCC-9. However, no differences (*p* > 0.05) were detected in the TP of Feta-type cheese made from control milk, 6%, and 9% protein MCC. The low TP content in Feta-type cheese made from MCC-3 could be due to the high lactose content that resulted from the addition of milk permeate to this treatment. On the other hand, salt and ash were not significantly different (*p* > 0.05) in the Feta-type cheese made from milk and MCC solutions. There was a significant difference (*p* < 0.05) detected in the fat content of Feta-type cheese made from MCC solutions. The Feta-type cheese made from milk and MCC with 3% protein showed no differences in the fat content with 19.26% and 19.08%, respectively. The fat content of Feta-type cheese made from milk and MCC solution with 3% protein was in the range of Feta-type cheese indicated in other studies [39,40,41]. The fat content dropped with elevating protein content in MCC solutions to 12.59% and 7.46% in MCC solutions with 6% and 9% protein, respectively. This can be explained by the differences in the TP:fat ratio in the starting milk/solutions used to make the cheeses. As mentioned earlier, the TP:fat ratio was not standardized in all treatments used to make Feta-type cheese. The TP:fat ratio in the final Feta-type cheese followed a similar trend as in the starting solutions. The ratio of TP:fat in Feta-type cheese made using whole milk and MCC with 3% protein was ~ 1.0. This ratio was 1.4 and 2.5 in Feta-type cheese made from MCC with 6% and 9% protein, respectively. The TS and TP of Feta-type cheese made from MCC were similar to that in typical Feta-type cheese (30–50% and 13–20%, respectively), as reported in previous studies [4,5,6,42,43,44,45]. No differences were found in the salt and ash contents of Feta-type cheese (approximately 11% and 12%, respectively). The salt and ash were high due to the high salt content in the brine [42,46]. Altan’s study reported that brining Feta-type cheese in 23% salt (sodium chloride) solution for 24 h resulted in approximately 10% salt in the final cheese, which is reasonable compared to our results [46]. The high salt content in the brine led to an increase in the salt and ash contents in Feta-type cheese [42]. We understand that the salt content in our study is higher than typical values for such cheeses because of the protocol we followed. The salt content will be adjusted to values found in commercial products in future studies.

The pH of Feta-type cheese made from milk and MCC solutions with 3%, 6%, and 9% protein is presented in Figure 1. The pH of Feta-type cheese after storing for 24 h in the brine solution was around 4.7 in control and MCC-3; however, the pH was 4.9 and 5.3 in MCC-6 and MCC-9, respectively. Feta-type cheese made from MCC-6 and MCC-9 had a higher pH because of the high buffering capacity due to increased protein content in those treatments [47]. The pH of Feta-type cheese made from MCC was similar to other studies that ranged from 4.6 to 5.6 [4,5,6,42,44,45,48]. However, the high pH in Feta-type cheese made from MCC-9 can decrease during storage or ripening because of the continued activity of lactic acid bacteria. It has been found that the pH of Feta-type cheese dropped from approximately 5.3 to 4.6 during 60 d of storage [6]. Other researchers reported that the pH of Feta-type cheese made from whole milk (3.0% fat) decreased from 5.07 to 4.57 during 6 months of storage [5]. Another study mentioned that the pH of Feta-type cheese made from milk protein concentrate solution with 12.0% protein decreased from 5.02 to 4.87 during 30 d of storage [49]. 

### 3.2. Yield and Hardness

The yield and adjusted yield of Feta-type cheese produced from MCC are indicated in Table 4. As expected, the yield and adjusted yield increased significantly (*p* < 0.05) with increasing protein content in MCC utilized in making Feta-type cheese. The yield of Feta-type cheese made from milk, 3%, 6%, and 9% protein MCC was approximately 17.5%, 19.0%, 36.6%, and 54.8%, respectively. While the adjusted yield was 19.2% in Feta-type cheese made from milk (adjusted to 55.0% moisture and 10.0% salt) compared to 21.4%, 37.2%, and 56.4% in Feta-type cheese made from 3%, 6%, and 9% protein MCC, respectively.

A significant difference (*p* < 0.05) was detected in the yield and adjusted yield of Feta-type cheese made from milk and MCC solutions. The yield and adjusted yield of Feta-type cheese made from milk and 3% protein MCC were not different (*p* > 0.05), although there was a slight increase in these values in Feta-type cheese made from 3% protein MCC. This can be related to the slight increase in the recovery of solids and mineral solids content of MCC-3 relative to milk [5,49,50]. Katsiari and Voutsinas found that decreasing the TS from 17.36% to 13.95% resulted in a reduction in the yield from 31.78% to 26.57% and adjusted yield from 27.00% to 18.67% [5] using 55% moisture as the desired water content. The yield and adjusted yield values of control and MCC-3 are in the range reported in other studies [5,48]. The yield and adjusted yield of Feta-type cheese produced from MCC-6 and MCC-9 were higher (*p* < 0.05) compared to the control and MCC-3, with MCC-9 having the highest (*p* < 0.05) yield and adjusted yield among all treatments. Increasing the protein content in MCC led to increased recovery of milk solids and minerals in Feta-type cheese, which resulted in elevating the yield and adjusted yield. Baig et al. [50], in their study on camel-milk feta, reported that elevating the ratio of casein:fat from 0.6 to 0.9 in camel milk resulted in increasing the cheese yield from approximately 7.8 to 9.0 [50]. As indicated earlier, the TP:fat progressively increased in our treatments, which would explain the higher yield and adjusted yield that was obtained as the protein content of MCC to make the cheeses was increased. The yield and adjusted yield of Feta-type cheese made from MCC with 6% and 9% protein were higher than previous studies that used milk or milk protein concentrate in making Feta-type cheese [5,49,51], which makes MCC with a higher protein content (≥6%) a suitable product to make Feta-type cheese for manufactures looking to improve cheese make efficiency. 

The hardness of Feta-type cheese produced from 3%, 6%, and 9% protein MCC is shown in Table 4. The hardness of Feta-type cheese ranged from 9.2 to 9.7 kg. No significant difference (*p* > 0.05) was detected in the hardness of Feta-type cheese made from milk compared to MCC-3, MCC-6, and MCC-9. The hardness of typical Feta-type cheese ranges from 1 to 8 kg, as reported in other studies [5,44,45,49,51]. The hardness of Feta-type cheese in our study is slightly higher, and this can be related to the high salt in brine solution used to store Feta-type cheese. It was found that elevating the salt concentration in brine resulted in increasing the hardness of Feta-type cheese [35]. Another study reported that the hardness of Gaziantep cheese increased from 3.31 to 52.5 N (0.33 to 5.35 kg) when the salt in brine was elevated from 5% to 25% [52]. It was found that the ratio of casein to fat did not affect the hardness of Feta-type cheese [44]. However, the hardness of Feta-type cheese made from MCC solutions is similar to the hardness of Feta-type cheese made from milk (control). 

### 3.3. Rheological Characteristics

**Figure 2:**
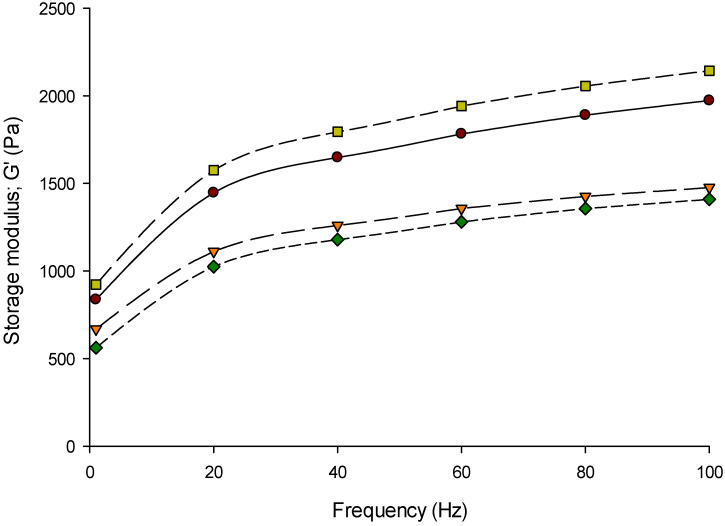
Elastic modulus (G′: Pa) of Feta-type cheese made from micellar casein concentrate (MCC). Treatment: Control (●) = Feta-type cheese made from whole milk; MCC-3 (▼) = Feta-type cheese made from MCC solution with 3% protein; MCC-6 (■) = Feta-type cheese made from MCC solution with 6% protein; MCC-9 (♦) = Feta-type cheese made from MCC solution with 9% protein measured at a range of 0.1 to 100 Hz frequency at 20 °C using dynamic rheological analysis (DSR).

**Figure 3:**
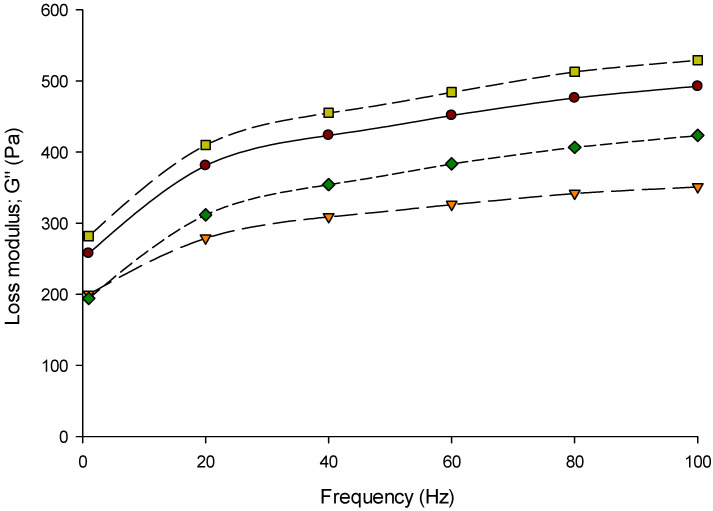
Viscous modulus (G′′: Pa) of Feta-type cheese made from micellar casein concentrate (MCC). Treatment: Control (●) = Feta-type cheese made from whole milk; MCC-3 (▼) = Feta-type cheese made from MCC solution with 3% protein; MCC-6 (■) = Feta-type cheese made from MCC solution with 6% protein; MCC-9 (♦) = Feta-type cheese made from MCC solution with 9% protein measured at a range of 0.1 to 100 Hz frequency at 20 °C using dynamic rheological analysis (DSR).

**Figure 4:**
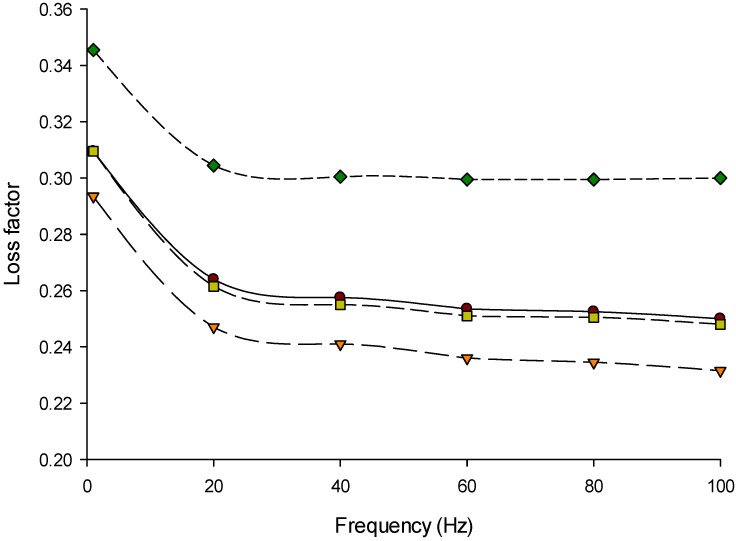
Loss factor (tan *δ = G*′′/G′) of Feta-type cheese made from micellar casein concentrate (MCC). Treatment: Control (●) = Feta-type cheese made from whole milk; MCC-3 (▼) = Feta-type cheese made from MCC solution with 3% protein; MCC-6 (■) = Feta-type cheese made from MCC solution with 6% protein; MCC-9 (♦) = Feta-type cheese made from MCC solution with 9% protein measured at a range of 0.1 to 100 Hz frequency at 20 °C using dynamic rheological analysis (DSR).

The frequency sweep is the optimum oscillatory test to examine the elastic (G′) and viscous (G′′) behaviors of Feta-type cheese while applying strain or stress [53,54]. The storage modulus (G′), loss modulus (G′′), and loss factor (tan *δ*) of Feta-type cheese made from 3%, 6%, and 9% protein MCC were measured at a range of 0.1 to 100 Hz frequency at 20 °C using the DSR are presented in Figure 2, Figure 3 and Figure 4, respectively. The Feta-type cheese made from 6% protein was slightly higher than others, while 9% protein MCC resulted in slightly lower G′ values. However, the G′ values of Feta-type cheese made from milk and MCC solutions (3%, 6%, and 9% protein) did not show differences (*p* > 0.05) at a range of 0.1 to 100 Hz frequency (Figure 2). The G′′ followed the same trend as in G′, and no differences (*p* > 0.05) were detected in the G′′ of Feta-type cheese at a range of 0.1 to 100 Hz frequency (Figure 3). On the other hand, the tan *δ* values of Feta-type cheese made from milk and different MCCs was significant (*p* < 0.05); thus, Feta-type cheese made with 9% protein MCC showed the highest tan *δ* values while Feta-type cheese made from 3% protein was the lowest at a range of 0.1 to 100 Hz frequency (Figure 4).

The G′ (Figure 2) and G′′ (Figure 3) of all Feta-type cheese samples increased (*p* < 0.05) with increasing frequency. However, the G′ was higher than G′′ in Feta-type cheese made from milk and MCC. This indicates that the Feta-type cheese has elastic behavior (gel) more than viscous behavior (liquid) [34,55,56]. The loss factor (tan δ) of Feta-type cheese made from milk, 3%, 6%, and 9% protein MCC is presented in Figure 4. There was a correlation between the loss factor with G′ and G′′. As a result, the loss factor can change according to the ratio of G′ and G′′. The loss factor of all Feta-type cheese samples ranged from 0.2 to 0.4.

The trends of G′ and G′′ were similar to other studies [34,57]. The G′ in Feta-type cheese made from MCC-6 was the highest, followed by Feta-type cheese made from milk and MCC-3, while the lowest G′ was noticed in Feta-type cheese made from MCC-9. The G′′ followed the same trend as in G′, except Feta-type cheese made from 9% protein showed higher values of G′′ than Feta-type cheese made from 3% protein MCC. These changes in G′ and G′′ of Feta-type cheese made from milk and MCC can be related to the changes in the composition of initial materials, cheese, or its structural changes [56,57]. The tan δ results are more consistent during measuring the loss and storage modulus at a frequency range. As a result, tan δ changed in the range of 0.2 to 0.4. It was found that the range of tan δ in soft cheeses was between 0.2 and 0.6 [34,56], which is similar to our results.

### 3.4. Color Analysis

The color of Feta-type cheese made from MCC is illustrated in Table 5. The Feta-type cheese samples had whitish, greenish, and yellowish colors. There were significant differences (*p* < 0.05) in the colors of Feta-type cheese made from MCC. The whiteness of Feta-type cheese made from milk (94.5) and MCC-3 (94.0) was significantly higher (*p* < 0.05) relative to Feta-type cheese samples made from MCC-6 (92.8) and MCC-9 (91.8). Feta-type cheese produced from 6% protein MCC (MCC-6) was more greenish (*p* < 0.05), while Feta-type cheese made from MCC-9 had the lowest (*p* < 0.05) green color relative to other Feta-type cheese samples. The yellow color was less in Feta-type cheese made from milk, as well as MCC-6 and MCC-9 compared to Feta-type cheese made from MCC with 3% protein. On the other hand, no visible changes (ΔΕ) were noticed in the color of Feta-type cheese.

The color of any dairy product is important because it is the key factor to consumers. The color of Feta-type cheese was relatively similar to that reported in other studies [6]. The high salt content (low ionic radius) in the brine led to increasing the hardness of Feta-type cheese [46], and this led to increased light reflection that increased the whiteness of cheese [6,58]. The high green and yellow color in Feta-type cheese made from MCC-3 and MCC-6 could be related to the milk permeate used to standardize the solids content. Thus, a higher amount of milk permeate was added to MCC-3 and MCC-6 formulations compared to MCC-9. As a result, Feta-type cheese made from 9% protein MCC had the lowest green and yellow colors, and this could be due to the low milk permeate added to the formulations of that treatment.

## 4. Conclusions

MCC solutions with different protein content (3.0–9.0%) were successfully used to make Feta-type cheese. The yield and adjusted yield of Feta-type cheese made from MCC solution with 6.0% and 9.0% protein were higher compared to Feta-type cheese made from milk. The hardness of Feta-type cheese produced from milk or MCC solutions was similar. The whiteness of Feta-type cheese was low as the protein content in the MCC solution elevated. While the yellowish and greenish colors were low in Feta-type cheese made from MCC 9.0% protein, no visible differences were observed in the overall cheese color. The rheological characteristics also improved in Feta-type cheese produced from MCC with 6% protein. There are several benefits of making Feta-type cheese from MCC over milk. First is the reduction in amounts of whey that is produced as a byproduct of making Feta-type cheese from milk. Additionally, using MCC as the starting material will offer flexibility to cheese manufacturers to develop a variety of Feta and Feta-type cheeses with different fat sources (milk fat or vegetable fat) and different fat levels, consequently offering a gamut of choices in terms of taste and nutrition to the changing needs of today’s consumer. Finally, MCC can be utilized in the manufacture of Feta-type cheese with a higher yield compared to milk, especially with 6% and 9% protein MCC with minimal impact on its textural and visual properties.

## Figures and Tables

**Table 1:** Mean (n = 3) values of micellar casein concentrate (MCC) formulations made for making Feta-type cheese.

Ingredients (%)	Treatment ^1^		
	MCC-3	MCC-6	MCC-9
Water	80.60	80.60	80.60
MCC powder	3.10	6.90	10.70
Milk permeate powder	8.20	4.50	0.80
Milk cream (40%)	8.10	8.00	7.90
Total	100	100	100

^1^ Treatment: MCC-3 = MCC solution with 3% protein; MCC-6 = MCC solution with 6% protein; MCC-9 = MCC solution with 9% protein.

**Table 2:** Mean (n = 3) composition values (% by weight) of whole milk and micellar casein concentrate (MCC) solutions utilized in the manufacture of Feta-type cheese.

Treatment ^1^	Composition ^2^				
	TS	TP	TP:Fat	Ash	NCN
Control	12.36 ^b^	3.01 ^d^	0.91 ^c^	0.72 ^b^	0.94 ^a^
MCC-3	14.72 ^a^	3.28 ^c^	0.99 ^c^	0.82 ^a^	0.61 ^d^
MCC-6	14.57 ^a^	6.39 ^b^	1.93 ^b^	0.83 ^a^	0.73 ^c^
MCC-9	14.83 ^a^	9.61 ^a^	2.91 ^a^	0.82 ^a^	0.87 ^b^
SEM	0.30	0.80	0.25	0.01	0.04
*p*-value	<0.05	<0.05	<0.05	<0.05	<0.05

^a–d^ Means in the same column not sharing a common superscript are different (*p* < 0.05). ^1^ Treatment: Control = whole milk; MCC-3 = MCC solution with 3% protein; MCC-6 = MCC solution with 6% protein; MCC-9 = MCC solution with 9% protein. ^2^ TS = total solids; TP = total protein; NCN = noncasein nitrogen.

**Table 3:** Mean (n = 3) composition values (% by weight) of Feta-type cheese made from micellar casein concentrate (MCC).

Treatment ^1^	Composition ^2^					
	TS	TP	Fat	TP:Fat	Salt	Ash
Control	49.03 ^ab^	17.36 ^b^	19.26 ^a^	0.90 ^c^	11.20	11.81
MCC-3	50.54 ^a^	14.04 ^c^	19.08 ^a^	0.73 ^c^	11.15	11.75
MCC-6	44.67 ^b^	18.08 ^ab^	12.59 ^b^	1.44 ^b^	11.33	12.36
MCC-9	45.47 ^ab^	19.10 ^a^	7.46 ^c^	2.55 ^a^	11.28	12.21
SEM	1.00	0.60	1.86	0.21	0.09	0.13
*p*-value	0.09	<0.05	<0.05	<0.05	0.92	0.34

^a–c^ Means in the same column not sharing a common superscript are different (*p* < 0.05). ^1^ Treatment: Control = Feta-type cheese made from whole milk; MCC-3 = Feta-type cheese made from MCC solution with 3% protein; MCC-6 = Feta-type cheese made from MCC solution with 6% protein; MCC-9 = Feta-type cheese made from MCC solution with 9% protein. ^2^ TS = total solids; TP = total protein.

**Table 4:** Mean (n = 3) yield (%), adjusted yield (%), and hardness (kg) values of Feta-type cheese made from micellar casein concentrate (MCC).

Treatment ^1^	Yield (%)	Adjusted Yield ^2^ (%)	Hardness (kg)
Control	17.54 ^c^	19.22 ^c^	9.25
MCC-3	19.04 ^c^	21.36 ^c^	9.67
MCC-6	36.61 ^b^	37.24 ^b^	9.60
MCC-9	54.77 ^a^	56.45 ^a^	9.44
SEM	4.60	4.50	0.18
*p*-value	<0.05	<0.05	0.42

^a–c^ Means in the same column not sharing a common superscript are different (*p* < 0.05). ^1^ Treatment: Control = Feta-type cheese made from whole milk; MCC-3 = Feta-type cheese made from MCC solution with 3% protein; MCC-6 = Feta-type cheese made from MCC solution with 6% protein; MCC-9 = Feta-type cheese made from MCC solution with 9% protein. ^2^ Yield adjusted to 55% moisture and 10% salt.

**Table 5:** Mean (n = 3) Hunter color (L*, a*, and b*) values of Feta-type cheese made from micellar casein concentrate (MCC).

Treatment ^1^	Hunter color			
	L*	a*	b*	ΔE
Control	94.55 ^a^	−1.31 ^b^	8.80 ^b^	-
MCC-3	93.97 ^a^	−1.35 ^bc^	10.55 ^a^	1.92
MCC-6	92.79 ^b^	−1.63 ^c^	10.21 ^ab^	2.37
MCC-9	91.82 ^b^	−1.02 ^a^	8.76 ^b^	2.89
SEM	0.40	0.07	0.30	0.20
*p*-value	<0.05	<0.05	<0.05	0.31

^a–c^ Means in the same column not sharing a common superscript are different (*p* < 0.05). ^1^ Treatment: Control = Feta-type cheese made from whole milk; MCC-3 = Feta-type cheese made from MCC solution with 3% protein; MCC-6 = Feta-type cheese made from MCC solution with 6% protein; MCC-9 = Feta-type cheese made from MCC solution with 9% protein.

## Data Availability

Not applicable.
